# The Safety of Ultrasound Contrast Agents in Children

**DOI:** 10.3390/diagnostics16060923

**Published:** 2026-03-20

**Authors:** Ruiqi Wang, Juan Wang, Hongli Zhai, Jingyu Chen, Ting Wang, Yi Tang

**Affiliations:** 1 Department of Ultrasound, Children’s Hospital of Chongqing Medical University, National Clinical Research Center for Child Health and Disorders, Ministry of Education Key Laboratory of Child Development and Disorders, Chongqing Key Laboratory of Pediatric Metabolism and Inflammatory Diseases, Chongqing 400014, China; rich199812@163.com (R.W.);; 2 Department of Ultrasound Medicine, Renji Hospital, School of Medicine, Chongqing University (The Fifth People’s Hospital of Chongqing), Chongqing 400044, China

**Keywords:** contrast-enhanced ultrasound, security, adverse reactions, ultrasound contrast agent, children

## Abstract

**Background/Objectives:** Retrospective analysis of the safety of contrast-enhanced ultrasound in children. **Methods:** A retrospective analysis was conducted on children who underwent contrast-enhanced ultrasound (CEUS) examinations at the Children’s Hospital of Chongqing Medical University from 2019 to 2025. Adverse reactions were classified into three grades (mild, moderate, and severe) based on severity. Following the pharmacological classification, the types of adverse reactions are divided into “Type A reactions” and “Type B reactions” and further classified into immediate and delayed reactions according to the timing of occurrence. **Results:** This study included 1604 pediatric patients who underwent a total of 1924 intravenous CEUS examinations. Seven patients experienced adverse reactions, yielding an overall adverse reaction incidence rate of 0.436% (95% Confidence Interval: 0.18–0.90%). Among these, six reactions were mild (6/7, 85.71%), and one was moderate (1/7, 14.29%). Among the seven patients, 16 clinical manifestations were recorded, comprising 12 type B reactions (12/16, 75%) and 4 type A reactions (4/16, 25%). Furthermore, all adverse reactions were classified as immediate onset. **Conclusions:** Under the conditions of this study, CEUS demonstrated good safety in the pediatric population, with no serious adverse events observed.

## 1. Introduction

In the pediatric population, CEUS provides greater precision than conventional ultrasound in assessing blood flow, perfusion, and lesion characterization. This can potentially reduce the need for additional examinations for pediatric patients [1,2].

Recently, the issue of radiation exposure in children has received significant attention. Although computed tomography (CT) offers unique diagnostic benefits, it also leads to an increased risk of exposure [3,4]. In contrast, magnetic resonance imaging (MRI) is similar to ultrasound in that it is free from radiation and provides excellent soft tissue imaging [5]. However, it is important to note that MRI is often expensive and requires sedation for pediatric examinations.

Currently, the primary contrast agent employed in CEUS is SonoVue (Product Name: SonoVue^®^; Manufacturer: Bracco Imaging SpA), a second-generation agent predominantly composed of stable microbubbles made from sulfur hexafluoride (SF6). This agent is metabolized through the lungs and demonstrates no nephrotoxicity.

The European Federation of Societies for Ultrasound in Medicine and Biology (EFSUMB) has issued guidelines regarding the use of contrast agents in CEUS [6,7]. In 2016, the U.S. Food and Drug Administration (FDA) approved the intravenous administration of SonoVue for imaging focal liver lesions in pediatric patients. While this application has yet to be approved in several European countries, it has significantly advanced pediatric CEUS technology globally [8]. In recent years, the application of CEUS in pediatrics has expanded from liver assessments to a wider range of clinical scenarios. Notably, Contrast-Enhanced Voiding Urosonography (ceVUS) has shown significant value in diagnosing vesicoureteral reflux in children. Compared to traditional Voiding Cystourethrography (VCUG), ceVUS not only provides higher diagnostic sensitivity but, more importantly, eliminates exposure to ionizing radiation [9], which is particularly critical for pediatric patients who require multiple follow-up evaluations.

The safety and efficacy of CEUS contrast agents administered intravenously in adults have been widely recognized [10,11,12]. However, relatively few large-sample studies have been conducted at home or abroad regarding CEUS via intravenous administration in children. Limited literature has investigated the correlations among the number of CEUS examinations, types of adverse reactions, timing, age, and other factors. This retrospective study aims to comprehensively evaluate the safety of intravenous CEUS in children aged 18 years and younger.

## 2. Methods

### 2.1. Study Design

This retrospective study has been approved by Children’s Hospital of Chongqing Medical University (approval numbers: CHCMU-XJS-2019-20; Date: 12 March 2019) and written informed consent forms have been obtained from the guardians of all participants. Informed consent was obtained from all subjects involved in the study.

### 2.2. Research Subjects

**Figure 1:**
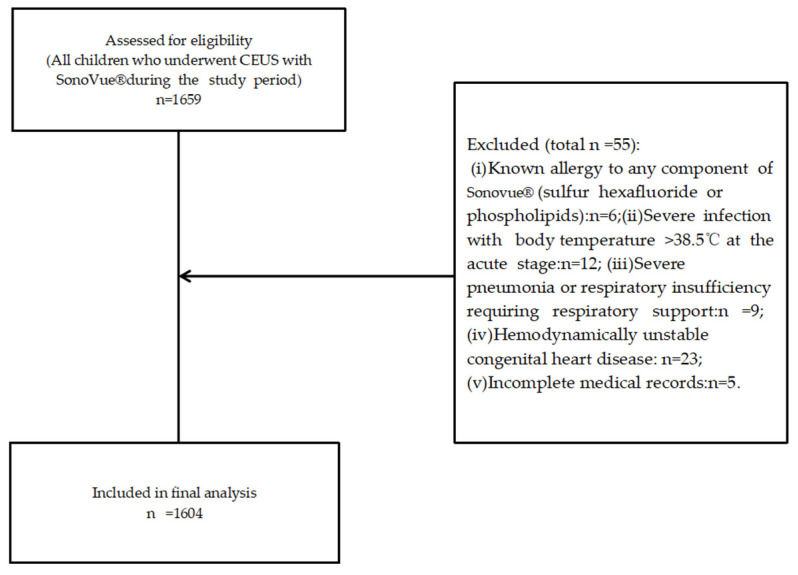
Research subject screening flowchart.

A retrospective analysis was conducted on 1604 pediatric patients enrolled at Chongqing Medical University Children’s Hospital between July 2019 and December 2025 (Figure 1), who collectively underwent 1924 intravenous injections of sulfur hexafluoride microbubble contrast-enhanced ultrasound examinations.

Among them, some children underwent multiple examinations due to the needs of clinical follow-up, (such as tumor efficacy assessment and postoperative follow-up). The imaging sites included the liver, kidneys, breasts, thyroids, uteruses, lymph nodes, and tumor metastatic foci.

#### 2.2.1. Inclusion Criteria

(i) Age 0~18 years old (including newborns and infants). (ii) Received intravenous injection of Sonovue for enhanced ultrasound examination during the study period.

#### 2.2.2. Exclusion Criteria

(i) For those with a known allergy to any component in Sonovue (such as sulfur hexafluoride or phospholipids). (ii) For acute-stage children with severe infections and a body temperature exceeding 38.5 °C. (iii) For children with severe pneumonia or respiratory dysfunction requiring respiratory support. (iv) For children with unstable hemodynamics and congenital heart disease. (v) For those who have not obtained the signed informed consent from their parents or legal guardians. (vi) The patient’s clinical data (such as examination records, follow-up information, etc.) are incomplete and cannot be analyzed effectively.

### 2.3. Contrast-Enhanced Ultrasound Imaging Protocol

Following the routine ultrasound examination, contrast-enhanced ultrasound was performed. Prior to the procedure, thorough communication was conducted with the child’s guardian, explaining the operational protocol and precautions. Written informed consent was obtained upon the guardian’s agreement. Throughout the examination, family members and clinical staff remained present. Emergency equipment was positioned adjacent to the ultrasound machine, with the trolley stocked with adrenaline, atropine, and dexamethasone sodium phosphate.

#### 2.3.1. Contrast Agents and Administration Regimens

This retrospective study utilized the second-generation contrast agent SonoVue (Product Name: SonoVue^®^; Manufacturer: Bracco Imaging SpA, Milano, Italy), composed of sulfur hexafluoride (SF6) microbubbles. The dosage employed was the FDA-recommended pediatric dose: 0.03 mL/kg, with a maximum single-examination dose not exceeding 2.4 mL. This dosage is applicable to children of all ages (including neonates and infants) and requires no adjustment based on age or examination indication [13].

#### 2.3.2. Injection and Image Acquisition

Draw the calculated dose of contrast agent into an empty syringe. Prior to injection, add 5 mL of 0.9% saline solution to the contrast agent vial, shake vigorously to ensure thorough mixing, and set aside. Select the optimal imaging plane for lesion visualization and stabilize the probe position. Employ real-time harmonic contrast imaging in dual-display mode with a mechanical index (MI) < 0.1. Rapidly inject the contrast agent through the intravenous infusion line (completed within approximately 2–3 s), followed immediately by flushing the line with 5 mL of 0.9% saline solution to ensure complete entry of the contrast agent into the bloodstream. Initiate the timer concurrently with contrast agent injection, continuously observing the contrast enhancement pattern within the lesion until complete clearance of the contrast agent. The entire examination process lasts 3~6 min, with all dynamic and static images preserved. All images are interpreted by a senior (over 10 years’ experience) sonographer qualified in contrast-enhanced ultrasound.

#### 2.3.3. Safety Monitoring and Repeated Checks

Throughout the procedure, closely monitor the patient for any abnormal clinical manifestations or signs. Should secondary or multiple intravenous ultrasound contrast examinations be required, ensure an interval of at least 20 min between injections to permit complete clearance of the previous contrast agent and provide an adequate safety observation window.

### 2.4. Definition and Classification of Adverse Reactions

#### 2.4.1. Definition of Adverse Reactions

Any unintended and harmful reaction to a drug, where there is at least a reasonable possibility of a causal relationship between the drug use and the adverse event [14].

#### 2.4.2. Criteria for Judging Adverse Reactions

(i) All suspected adverse events were independently reviewed and confirmed by two experienced pediatric ultrasound physicians with CEUS qualifications based on medical records. (ii) Judgments were made after comprehensive and prudent consideration only when clinical manifestations were clear and only CEUS contrast agent injection was administered during the observation period. (iii) For cases where underlying diseases might cause similar symptoms, the correlation with the contrast agent was determined through discussion.

The incidence rate of adverse reactions = Number of patients experiencing adverse reactions ÷ Total number of examined patients × 100% (For patients undergoing multiple examinations: If an adverse reaction occurs after any single examination, that patient is counted in the adverse reaction group; if the same patient experiences adverse reactions after multiple examinations, they are still counted as one patient).

#### 2.4.3. Grading of Adverse Reaction Severity

Mild: Self-limiting reaction, with mild symptoms, requiring no treatment or only supportive treatment based on symptoms (such as bedside observation, oral antihistamines). Includes localized urticaria, mild nausea, transient headache, etc.

Moderate: The symptoms are obvious and require medical intervention, but there are no immediate life-threatening manifestations. This includes generalized urticaria, bronchospasm (responsive to oxygen inhalation or bronchodilators), tachycardia, severe vomiting, etc.

Severe: Life-threatening, requiring urgent rescue intervention. This includes severe hypotension, laryngeal edema, sudden cessation of breathing, sudden cardiac arrest, loss of consciousness, etc. [15,16].

#### 2.4.4. Classification of Adverse Reactions

Since this was a retrospective observational study, serum trypsin-like enzyme, skin, and specific IgE antibody tests were not conducted on patients who experienced adverse reactions. Therefore, it was impossible to distinguish between allergic and physiological reactions from an immunological perspective. Thus, this study adopts the general pharmacological terminology for the classification of adverse reactions [17], namely the A/B reaction classification framework (Type A reaction: dose-dependent, predictable, and related to the pharmacological effects of the drug. Type B reaction: non-dose-dependent, unpredictable, and specific to the patient).

#### 2.4.5. Characteristics of the Time of Occurrence of Adverse Reactions

They are classified into immediate reactions (occurring within 1 h after administration, usually within 5 min) and delayed reactions (occurring from 1 h to several days after administration).

#### 2.4.6. Safety and Outcome of Adverse Reactions

To ensure comprehensive identification of adverse reactions, we have implemented the following monitoring measures: (i) In-hospital real-time monitoring: During the CEUS examination and for at least 30 min afterward, healthcare personnel continuously monitor vital signs (heart rate, respiration, and oxygen saturation) at the bedside and observe for any abnormal clinical manifestations or symptoms in all pediatric patients. (ii) Post-discharge monitoring for delayed reactions: To identify delayed reactions, guardians of all pediatric patients are provided with verbal and written instructions to closely monitor the child for 24–72 h after the examination for any abnormal manifestations, such as rash, fever, nausea, vomiting, or difficulty in breathing, and are provided with a 24-h contact number for prompt reporting. (iii) Confirmation and Outcome of Adverse Reactions: All suspected adverse reaction events are double-confirmed through medical records, telephone follow-ups, or follow-up visit records. The child’s prognosis is documented, including complete resolution of symptoms, the need for in-hospital observation, residual sequelae, or death.

#### 2.4.7. Subgroup Descriptive Analysis

This study analyzes the occurrence of adverse reactions across various clinical characteristic subgroups, including tumor and non-tumor patients, as well as single-dose and multiple-dose users. However, due to the small total number of adverse events observed, only a descriptive presentation of the findings is provided, and inter-group comparisons are not conducted.

### 2.5. Statistical Analysis

Statistical analyses were conducted using SPSS software (version 22.0, IBM (Armonk, NY, USA)). Normality tests were performed on all continuous variables, utilizing the Shapiro–Wilk test to evaluate the adherence of the data to a normal distribution. Measurement data that followed a normal distribution were reported as mean ± standard deviation (Mean ± SD), whereas non-normally distributed measurement data were expressed as median and interquartile range (IQR, 25th–75th percentiles). Categorical data were represented as frequencies and percentages (*n*, %). Comparisons of patient characteristics between groups for categorical variables were carried out using chi-square tests or Fisher’s exact tests. The confidence interval is calculated using the Clopper–Pearson exact method. All tests were two-sided, with a significance threshold set at *p* < 0.05.

## 3. Results

### Characteristics of the Research Population

During the study period, a total of 1604 pediatric patients met the inclusion criteria (Figure 1), undergoing 1924 intravenous injections of sulfur hexafluoride microbubble-enhanced CEUS. Some patients required multiple examinations (e.g., tumor response assessment or postoperative follow-up). The cohort comprised 742 males (46.26%) and 862 females (53.74%). The mean age was 8.31 years (range: 15 days to 18 years), with the age distribution predominantly concentrated in the 0–3 years (657 cases, 40.96%) and 3–6 years (462 cases, 28.80%) age groups.

The abdomen was the most frequently examined anatomical region (1032 out of 1604, 64.34%), with the liver identified as the predominant target organ (844 out of 1604, 52.62%). The majority of indications were associated with space-occupying lesions. A total of seven adverse reactions were recorded (Table 1), resulting in an overall incidence rate of 0.436% (95% CI: 0.18–0.90%). These reactions included six mild cases (6 out of 7, 85.71%) and one moderate case (1 out of 7, 14.29%). Adverse reactions were most commonly observed in the 0–3 years age group (4 out of 7, 57.14%). No statistically significant differences were found in the incidence of adverse reactions across different age groups and genders (Table 2).

As illustrated in Table 3, among the seven children who experienced adverse reactions, there was no statistically significant difference in the incidence of adverse reactions between the tumor and non-tumor groups (*p* > 0.05). Additionally, no statistically significant differences were noted within the subgroups of mild, moderate, and severe adverse reactions (all *p* > 0.05).

The detailed signs and symptoms of adverse reactions in pediatric patients are presented in Table 4. According to the pharmacological classification (Table 5), a total of 16 clinical manifestations were observed in 7 pediatric patients experiencing adverse reactions: 12 (75%) were classified as type B reactions, while 4 (25%) were classified as type A reactions. Among the mild type B reactions, there were five instances of choking cough, two instances of localized urticaria, and one instance of local angioedema. Mild type A reactions included three instances of limited nausea or vomiting and one instance of transient flushing, fever, or chills. Moderate type B reactions encompassed four instances of wheezing or bronchospasm accompanied by mild hypoxia. There were no recorded instances of severe type B reactions. The most common type B reaction was choking cough (*n* = 5). Among type A reactions, limited nausea or vomiting was most frequently observed (*n* = 3). Additionally, one pediatric case with a history of repeated CEUS examinations exhibited two adverse reactions, both presenting as retching type A reactions. All reported adverse reactions in children were immediate-onset type in terms of occurrence timing.

Additionally, the study found (Table 6) that there was no statistically significant difference in the incidence of adverse reactions between single-dose administration and multiple-dose administration (≥2 times) in pediatric patients (all *p* > 0.05).

## 4. Discussion

In 2016, the FDA approved the use of Sonovue intravenously for imaging focal liver lesions in children, marking a significant milestone in the global development of pediatric contrast-enhanced ultrasound. However, non-hepatic applications in children remain off-label uses [6,7,8]. However, it is important to note that off-label usage does not equate to “non-compliant” usage. Rather, it refers to applications conducted under strict adherence to clinical standards and indications. Clinical practice has supported expanding off-label applications in pediatrics, such as those involving the kidneys, spleen, and pancreas. Accumulating evidence has confirmed the diagnostic value of these applications [18,19,20,21]. These applications have demonstrated distinct advantages, particularly in diagnosing and treating critically ill children [22].

In this study group, the incidence of adverse reactions in pediatric patients was 0.436% (7/1604), which is consistent with the reported incidence of adverse reactions in adults undergoing CEUS [10,15,23]. The majority of symptoms were mild and self-limiting (85.71%, 6/7), requiring only symptomatic treatment for complete resolution. Only one case (14.29%, 1/7) presented moderate adverse reactions, with vital signs rapidly stabilizing following active management.

However, some patients with adverse reactions exhibit multiple clinical symptoms and require close attention. It is recommended that emergency rescue equipment and medications (such as epinephrine and antihistamines) be placed near the examination room when conducting CEUS examinations to ensure a prompt response to possible acute severe reactions.

Of the seven children who experienced adverse reactions, only one had undergone two CEUS examinations, with identical type B reactions, such as retching, occurring both times. Studies [24,25] have shown that contrast agent-related adverse reactions differ from type B reactions. Type A reactions include symptoms such as dizziness, nausea, and vomiting. These reactions may be associated with vasovagal responses triggered by patient anxiety during the examination. This suggests that calming the child’s emotions prior to the procedure may reduce the likelihood of such reactions.

This study also found that increasing the frequency of administration did not significantly alter the risk of adverse reactions. Multiple administrations (including twice and three or more times) showed no significant difference in the incidence of adverse reactions compared to a single administration. There was also no significant difference between pediatric patients with tumorous and non-tumorous conditions. However, due to the limited number of observed adverse events, further validation in larger cohorts with higher event rates is required. Nevertheless, these findings provide new insights into the safety of CEUS for pediatric patients requiring repeated long-term CEUS examinations, particularly those with tumorous conditions.

In this study, all pediatric patients experienced immediate-onset adverse reactions, with over half occurring within 1 min (4/7, 57.14%). This may be associated with the rapid clearance of microbubble contrast agents, as Sonovue is almost completely eliminated via the respiratory system approximately 8 min after peripheral intravenous injection [26]. However, based on reports in pediatric literature [27,28], delayed allergic reactions are most commonly observed 2 to 3 h after testing. Consequently, in this study, we adopted a strategy combining 30 min of bedside monitoring in the hospital with active follow-up 24 to 72 h after discharge to comprehensively cover the time window for delayed reactions. We recommend that in clinical practice, particularly in outpatient settings, in addition to routine in-hospital observation, efforts should be made to strengthen guidance for guardians on recognizing delayed reactions and to provide clear reporting channels (such as a 24-h contact number) to effectively mitigate potential risks.

Additionally, it was observed that the highest incidence of adverse reactions occurred in the 0–3 years age group (4 out of 7, 57.14%). This phenomenon may be attributed to the relatively larger population size of this age group in the study. However, given the small sample size of children experiencing adverse reactions in this group, the correlation with patient age necessitates further investigation.

A comprehensive comparison of domestic and international literature [29,30,31] demonstrates that CEUS is safer than iodinated CT contrast agents. The recurrence rate of adverse reactions to iodinated contrast media is as high as 31.1% [29]. While some studies report the effectiveness of preoperative steroid use in reducing recurrence in patients with moderate-to-severe hypersensitivity reactions (HSRs), conclusive evidence remains elusive [29].

Furthermore, compared to gadolinium-based contrast agents used in MRI, CEUS contrast agents exhibit superior long-term safety [32]. CEUS uses an inert gas core that is metabolized and cleared quickly through pulmonary respiration. This characteristic allows for multiple intermittent bolus injections during a single examination without cumulative risk [33]. Additionally, CEUS demonstrates no hepatic or renal toxicity, making it suitable for special and critically ill patient populations [34]. Compared to gadolinium-based agents, CEUS is a safer alternative with regard to potential long-term deposition effects.

CEUS has gradually expanded from its initial application of evaluating focal liver lesions to multiple areas of pediatric imaging [9,10,11,12]. Robust safety data are crucial for supporting the continued use and potential expansion of CEUS as a diagnostic tool.

## 5. Limitations

This study acknowledges several limitations. Firstly, some pediatric patients may experience adverse events; however, the symptoms may be too mild to notice or could be mistakenly attributed to CEUS contrast agents in the absence of a causal relationship, both of which may introduce selection bias. Secondly, although this research was conducted at a tertiary Grade A hospital in China, the single-center design may result in regional bias. Consequently, multicenter safety analyses across diverse regions and encompassing a wider range of indications would provide more robust and convincing results.

## 6. Conclusions

This study summarizes the incidence of adverse events and their corresponding signs and symptoms, demonstrating that under the research conditions, CEUS exhibited good safety in the pediatric population, with no severe adverse events observed.

However, as a single-center retrospective study, limitations may exist in the form of underreporting of minor adverse reactions or omission of delayed events. Future prospective, multicenter studies are required to further accumulate safety data, thereby supporting broader clinical applications in pediatrics beyond the approved indications for intravenous CEUS.

## Figures and Tables

**Table 1:** Areas for CEUS examination.

	Frequency/Location	Total (Rate, %)	Severity Grading of Adverse Reactions		
			Mild	Moderate	Severe
Total	1604	7 (0.436)	6 (0.374)	1 (0.062)	
Abdomen	Liver	844		1 (0.062)	
	Pancreas, spleen, kidneys, and retroperitoneal masses, etc.	188	5 (0.312)		
Thyroid		94	0		0
Genitals	Ovaries, uterus, and testicles, etc.	178	1 (0.062)		0
Limbs	Osteosarcoma, etc.	135	0		0
Others	Lymph nodes, hemangiomas, and the presence of metastatic lesions after surgery, etc.	165	0		

**Table 2:** Incidence of adverse reactions among different gender and age groups.

	All Patients (*n*)	Percentage of People (%)	Patients with Adverse Reactions (*n*)	Incidence Rate of Adverse Reactions (%)	Chi-Square	*p* Value
Gender					0.884	0.347
Male	742	46.26	2	0.270		
Female	862	53.74	5	0.580		
Age group					2.831	0.725
0~3 y	657	40.96	4	0.609		
3~6 y	462	28.80	1	0.216		
6~9 y	113	7.04	0	0		
9~12 y	167	10.41	1	0.599		
12~16 y	97	6.05	1	1.031		
16~18 y	108	6.73	0	0		

**Table 3:** Analysis of the correlation between tumorous and non-tumorous lesions and the risk of adverse reactions.

	Total Patients (*n* = 1604)	Non-Tumoral Lesion (*n* = 465)	Tumoral Lesion (*n* = 1139)	*p* Value
Any Adverse Reaction *n* (%)	7	1	6	0.66
Mild *n* (%)	6	1	5	0.83
Moderate *n* (%)	1	0	1	0.64
Severe *n* (%)	0	0	0	1.00

**Table 4:** Descriptions of adverse reaction incidents.

	Description of Adverse Reaction Events (Seven Cases)	Clinical Symptoms (16 Items)
1	Female, four years and two months old. Diagnosed with hepatic hemangioma. After 1 min of injection, she experienced choking cough. Her complexion was flushed and her breathing was rapid. The blood oxygen saturation fluctuated between 85% and 88%. Immediately, she was given oxygen via a mask at the hospital’s sedation center, and dexamethasone and 10% calcium gluconate were intravenously injected. After 25 min, the symptoms were relieved.	Coughing, Flushed face, Mild hypoxia
2	Male, ten years old. Diagnosed with a spleen mass. After two minutes of injection, the patient experienced mild choking cough and local angioedema in the left eyelid. Immediately, oxygen inhalation and symptomatic treatment were provided at the sedation center of our hospital.	Coughing, Local angioedema,
3	Male, one year old. Diagnosed with right retroperitoneal neuroblastoma. After intravenous injection of contrast agent, symptoms such as coughing, vomiting, localized urticaria, and mild hypoxia occurred 30 s later. After receiving symptomatic supportive treatment such as suctioning and oxygen supply at the sedation center of our hospital, the patient’s vital signs were stable, with blood oxygen saturation maintained at 93–95%. There was no restlessness, choking cough, or vomiting. After the patient’s vital signs were stable, the doctor carried an oxygen cylinder and sent him back to the ward. There were no special discomforts during the follow-up later.	Cough, Vomiting, Localized urticaria, Mild hypoxia
4	A 14-year-old female was diagnosed with a right-sided serous cystadenoma, accompanied by extensive hemorrhage, and subsequently underwent surgical excision and reconstruction of the right fallopian tube. On 24 August 2022, she underwent her first CEUS examination. Thirty seconds after the injection of 2.4 mL of contrast agent, she experienced dry heaving without any vomiting. She spontaneously recovered after ten seconds, exhibiting stable vital signs and no symptoms of cyanosis, rash, or hypoxia. The resident physician from our hospital transported her back to the ward with supplemental oxygen. On 5 April 2023, she underwent a second CEUS examination. Within one minute of injecting 2.46 mL of contrast agent, she again exhibited gastrointestinal symptoms of dry heaving, which resolved immediately after 15 s. Her vital signs remained stable, and after remaining calm in the sedation center for 15 min, she was comprehensively assessed and transported back to the ward by the physician with an oxygen tank.	Vomiting
5	A female patient, one year and eight months old, was diagnosed with right retroperitoneal neuroblastoma. After 58 s of undergoing the administration of Sonovue, the child exhibited an acute adverse reaction, characterized by coughing, brief localized urticaria, and mild cyanosis of the lips. Blood oxygen saturation was measured at 88%. Immediate resuscitation was initiated at the sedation center, including symptomatic management with oxygen therapy. The child’s symptoms improved, and blood oxygen saturation was maintained at 95%. Once the vital signs stabilized, the physician transported the patient back to the ward with an oxygen cylinder. Follow-up showed no significant discomfort.	Coughing, Localized urticaria, Mild hypoxia
6	A 2-year and 11-month-old female was diagnosed with left retroperitoneal neuroblastoma. Approximately 45 s after intravenous administration of Sonovue 0.34 mL, she exhibited several episodes of coughing, slight cyanosis of the lips, and a mild decrease in mental status. Her blood oxygen saturation was measured at 89%. Following a rapid assessment by the physician, she was promptly taken to the sedation center with a blood pressure monitor. At the sedation center, symptomatic treatment including oxygen supplementation was provided. The patient’s symptoms improved, she became responsive, and her blood oxygen saturation rose to between 90 and 95%, with a restoration of normal lip color. After ensuring stable vital signs, the physician transported her back to the ward with an oxygen tank.	Coughing, Mild hypoxia
7	Female, one year and 11 months old. Diagnosed with right retroperitoneal neuroblastoma. After intravenous injection of Sonovue for 1 min, she experienced vomiting symptoms, which later subsided.	Vomiting

All descriptions of skin reactions are based on clinical observations and use standard terms in dermatology.

**Table 5:** The manifestations of A/B type reactions in children’s adverse reactions.

Mild				Moderate				Severe			
Type B Reaction		Type A Reaction		Type B Reaction		Type A Reaction		Type B Reaction		Type A Reaction	
Localized urticaria	2	Limited nausea or vomiting	3	Diffuse urticaria	0	Preventive nausea/vomiting	0	Diffuse edema, or facial edema accompanied by breathing difficulties	0	Vagal nerve response to treatment resistance	0
Limitation: “Itchy”/”Itchy Throat”	5	Headache/Dizziness/Anxiety/Changes in taste	0	Shortness of breath/Bronchospasm, mild or without hypoxia	4	Emergency state of hypertension	0	Shortness of breath/Bronchospasm, severe hypoxia	0	Arrhythmia	0
Local angioedema	1	Transient flushing/Fever/Chills	1	Facial edema without respiratory distress	0	Isolated chest pain	0	Anaphylactic shock	0	Convulsions, epilepsy	0

**Table 6:** Analysis of the correlation between dosing frequency and the risk of adverse reactions.

Administration Frequency	Total Patients (*n* = 1604)	Any Adverse Reaction (*n*, %)	Mild (*n*, %)	Moderate (*n*, %)	Severe (*n*, %)
Single (1 time)	1337	6 (0.449)	5 (0.374)	1 (0.075)	0
Twice (2 times)	214	1 (0.467)	1 (0.467)	0	0
Multiple (≥3 times)	53	0	0	0	0
*p* Value		1.000	1.000	1.000	1.000

## Data Availability

The original contributions presented in this study are included in the article. Further inquiries can be directed at the corresponding author.

## Abbreviations

The following abbreviations are used in this manuscript:

CEUS	Contrast-Enhanced Ultrasound
CT	Computed Tomography
MRI	Magnetic Resonance imaging
EFSUMB	The European Federation of Societies for Ultrasound in Medicine and Biology
FDA	Food and Drug Administration
ceVUS	Contrast-Enhanced Voiding Urosonography
VCUG	Voiding Cystourethrography
HSR	Hypersensitivity Reactions
CI	Confidence Interval
